# Factors Associated with Interruptions of Enteral Nutrition and the Impact on Macro- and Micronutrient Deficits in ICU Patients

**DOI:** 10.3390/nu15040917

**Published:** 2023-02-11

**Authors:** Arezina N. Kasti, Maria Theodorakopoulou, Konstantinos Katsas, Kalliopi D. Synodinou, Maroulla D. Nikolaki, Alice Efstathia Zouridaki, Stylianos Fotiou, Aliki Kapetani, Apostolos Armaganidis

**Affiliations:** 1 Department of Nutrition and Dietetics, Attikon University General Hospital, 12462 Athens, Greece; 2 1st ICU Department, Evangelismos Hospital, Intensive Care Medicine, 10676 Athens, Greece; 3 2nd ICU Department, Attikon University Hospital, Intensive Care Medicine, 12461 Athens, Greece; 4 Medical School, National and Kapodistrian University of Athens, 11527 Athens, Greece; 5 Department of Nutrition and Dietetics Sciences, Hellenic Mediterranean University, 72300 Crete, Greece; 6 Department of Human Biology and Health Studies, University of Toronto, Toronto, ON M5S, Canada; 7 Department of Nutrition and Dietetics, School of Health Sciences and Education, Harokopio University, 17676 Athens, Greece

**Keywords:** interruption of enteral nutrition, intensive care, caloric deficit, malnutrition, micronutrients, antioxidants

## Abstract

Background and Aim: Feeding interruptions in critical care patients are often unjustified. We aimed to determine the causes, duration, and frequency of enteral nutrition interruptions (ENIs) and to assess macronutrients and antioxidant deficits according to European Society of Parenteral Enteral Nutrition (ESPEN) guidelines. Methods: We prospectively enrolled Intensive Care Unit (ICU) patients admitted for more than 48 h with an inability to orally eat from April to December 2019. The type of enteral nutrition, the number of calories administered, the time of feeding initiation, the reasons for delaying feeding, and the causes for ENI were recorded. Results: 81 patients were enrolled, with a median duration of ENIs of 5.2 (3.4–7.4) hours/day. Gastric residual volume (GRV) monitoring—a highly controversial practice—was the most common cause of ENI (median duration 3 (2.3–3) hours/day). The mean energy intake was 1037 ± 281 kcal/day, while 60.5% of patients covered less than 65% of the total energy needs (1751 ± 295 kcal/day, according to mean Body Mass Index (BMI)). The median daily protein intake did not exceed 0.43 ± 0.3 gr/kg/day of the actual body weight (BW), whereas ESPEN recommends 1.3 gr/kg/day for adjusted BW (*p* < 0.001). The average administration of micronutrients and antioxidants (arginine, selenium, zinc, vitamins) was significantly less than the dietary reference intake (*p* < 0.01). Conclusion: ENIs lead to substantial caloric, protein, and antioxidant deficits.

## 1. Introduction

Critically ill patients need the utmost medical nutrition therapy and specialized feeding protocols because critical illness is associated with increased catabolic stress, systemic inflammatory responses and, therefore, energy and protein requirements [1]. Critical care patients usually undergo feeding interruptions for various reasons, such as diagnostic or therapeutic procedures, which often lead to malnutrition and adverse outcomes [2]. Underfeeding has been associated with a prolonged hospital length of stay, a high prevalence of complications, such as infections and organ failure, and high mortality rates [3,4,5]. The metabolic regulatory mechanisms are radically altered, resulting in a disproportionate release of cytokines and stress hormones, with a subsequent dysregulation of energy and protein metabolism, and micronutrient stores, thus exacerbating malnutrition and leading to a vicious circle [6].

Glucose is the preferred energy substrate in the acute phase of illness [7]. The glucose demanded by infection processing and wound or injury healing and the required amount for brain, renal, and hematopoietic system function are derived in part from proteolysis and lipolysis [8]. The production of glucose from amino acids and glycerol occurs via gluconeogenesis and the glycolysis pathway. In patients receiving enteral (EN) or parenteral nutrition (PN), endogenous glucose production was increased by approximately 150–210 g/day on day 4 and by 130–150 g/day on days 9–10 of ICU admission [7].

Lipids are used primarily due to their high caloric content as an energy-dense substrate, lowering the required number of carbohydrates and CO_2_ production as part of EN. Additionally, they provide the building blocks of the cell membrane structure and essential fatty acids, thereby preventing essential fatty acid deficiency and allowing for fat-soluble vitamins delivery [9]. During critical illness, significant changes emerge in endogenous lipid profiles [10]. Among others, a decline in lipid absorption and a rise in lipolysis result in increased plasma triglycerides and decreased plasma High-Density Lipoproteins (HDL) and Low-Density Lipoproteins (LDL) [11]. The magnitude of the change in the amount and composition of HDL seems to reflect the severity of inflammation and may affect the patient’s clinical outcome [12]. The increased synthesis and secretion of triglyceride-rich lipoproteins from the liver and cytokine-induced hyperlipoproteinemia, named ‘lipemia of sepsis’, were normally considered to represent the mobilization of lipid stores in order to boost the host’s response to infection [13]. Serum cholesterol levels tumble to almost 50%, specifically in severe sepsis [10]. These lipid abnormalities likely mediate organ dysfunction and failure. Thus, free fatty acids (FA) induce inflammation and have a toxic effect on tissues [11]. Metabolic abnormalities in severe infection are dramatic, so sepsis is considered an acquired disease of intermediary metabolism [14]. During septic conditions, lipolysis in peripheral tissues is further stimulated by rising FA levels, whose composition also alters with a decrease in polyunsaturated FA (PUFA) and an increased ratio of omega-6 to omega-3 FA [10].

Critical illness leads metabolism to a dynamic state divided into different stages. During the acute phase, the patient goes through the ebb phase, which includes the initial 24 to 48 h, and the flow phase, which lasts until seven days. After the acute phase, some patients may enter the recovery stage, while others remain in a prolonged critical illness. After the ebb phase, the catabolic response increases, and the human-stored components, including proteins, are reduced [15]. Skeletal muscle loss is the result of muscle protein breakdown to the detriment of protein synthesis. In critically ill patients, an imbalance between protein synthesis and breakdown rates results in a catabolic state, with a marked muscle loss of up to 1 kg per day over the first ten days of the ICU stay [16,17,18].

Oxidative stress, caused by an imbalance among increased reactive oxygen (reactive oxygen species, ROS), nitrogen species, and endogenous antioxidant mechanisms, is associated with protein and lipid oxidative damage and usually coexists with septic shock, severe pancreatitis, acute respiratory distress syndrome (ARDS), major burns, and trauma [19]. Antioxidants are compounds that prevent the transport of electrons from an organic molecule to another and to oxygen. [20] The antioxidant micronutrients, such as selenium, zinc, and vitamins A and C (ascorbic acid), belong to the primary antioxidant defense. In such inflammatory conditions, their circulating levels are decreased below reference ranges [18]. ICU patients are mostly a very heterogeneous population based on their illness. While the literature offers possible solutions to many issues with some degree of consensus, others, like the recommendations on micronutrients and vitamins, are still being discussed [21]. Margaritelis and his colleagues highlighted that most ICU patients developed severe antioxidant deficiency despite the different stress levels [16]. Similarly, Koekkoek et al. indicated a reduction in vitamin C, vitamin E, and selenium levels already during admission [17]. Ruocco and her colleagues highlighted that zinc and selenium deficiency occurs in critically ill patients as a response to oxidative stress [22]. Another study from Spain confirmed that ICU patients showed substantially lowered selenium levels during ICU admission, while these selenium levels further decreased after a week in the ICU [23]. Additionally, Hoffmann et al. mentioned that many critically ill patients suffer from vitamin D deficiency (serum 25-hydroxyvitamin D (25(OH)D) < 20 ng/mL), with levels beneath 12 ng/dL [21].

The primary aim of the present study is to determine the causes, duration, and frequency of ENI episodes and to assess the impact on macro- and micronutrient deficits (mainly antioxidants). The secondary endpoints will evaluate the nutrient intake in ICU patients fed with EN and examine the possibility of EN sufficiency in reaching the metabolic requirements of the critically ill without supplementation.

## 2. Materials and Methods

### 2.1. Subjects and Study Design

A single-center, prospective, observational study was performed in a mixed 19-bed ICU in a tertiary hospital (“Attikon” University Hospital) in Athens, Greece. Participants were enrolled between April and December 2019. Patients with an ICU stay of more than 48 h and an inability to feed orally (mostly mechanical ventilated patients or those under sedation (Supplementary Table S1)) were included in the present study. The exclusion criteria were as follows: patients <18 years of age, partial or exclusive oral food intake, total or peripheral parenteral nutrition, patients unable to be fed (with severe septic shock; hemodynamically unstable), and enrolled patients who were discharged or died between the 1st and 7th day of admission to the ICU.

### 2.2. Sample Size

The initial sample size was calculated to be 93 subjects, based on one of the primary aims to determine the proportion of patients with low energy intake (less than 65% of the total energy needs for each subject), with a confidence interval of 95%, expected subjects with a low energy intake of 60%, and a margin of error of 10%.

### 2.3. Data Collection

Demographic data, weight, and height were recorded upon admission to the ICU, and Body Mass Index (BMI) (weight (kg)/height (m^2^)) was calculated [24]. Patients with a BMI <18.5 kg/m^2^ were classified as underweight; those with a BMI from 18.5kg/m^2^ to <25 kg/m^2^ were classified as normal; those with a BMI from 25 kg/m^2^ to <30 kg/m^2^ were classified as overweight; and those with a BMI ≥30 kg/m^2^ were classified as obese [25]. Disease severity indicators (Acute Physiology, Age, Chronic Health Evaluation Score (APACHE II), and Sequential Organ Failure Assessment (SOFA) score) upon admission, the need for mechanical ventilation at the first week of ICU hospitalization, and the modified Nutrition Risk in Critically Ill (mNUTRIC) score were also recorded when the data were available [26].

### 2.4. Dietary Intake

Energy, macro-, and micronutrient daily intake (vitamins A, C, and D, selenium, manganese, zinc, omega-3 fatty acids, and arginine) were calculated based on the labels of commercial formulas (various types and brands) and were collected for a seven-day follow-up period. The adequacy of the macro- and micronutrient intake was assessed according to the most recent European Society for Clinical Nutrition and Metabolism (ESPEN) guidelines [27]. In the absence of indirect calorimetry and VO_2_ or VCO_2_ measurements, patients were prescribed EN with a simplistic weight-based value, targeted to achieve 25 kcal/kg (Ideal Body Weight) of energy and 1.2 g/kg of proteins daily [28]. The caloric load from propofol (1.1 kcal/mL) and 5% dextrose (4 kcal/g) was also taken into account [29,30].

The frequency, the causes, and the duration of ENIs were recorded. The causes of ENIs were classified into three groups: diagnostic procedures, interventional procedures, and patient-related factors. Diagnostic procedures included GRV management (measured by aspiration using a syringe) [31] and body imaging techniques (Computed Tomography (CT) scans, Magnetic Resonance Imaging (MRI), abdominal ultrasounds (fasting for optimal visualization)). The feeding protocol for patients prescribed EN included GRV monitoring three times a day. Interventional procedures included extubation/(re)intubation, surgeries, percutaneous endoscopic gastrostomy (PEG) tube placement, drainages, and physiotherapies. Patient-related factors included gastrointestinal dysfunctions (high GRV, diarrhea, and bowel ischemia), pleural effusions, and secretions. ENIs were retrieved from the medical records (ICU charts).

Nutritional and ENIs data were collected from the first day of admission. However, the average nutrition intake and the duration of ENIs recorded in the first two days of the ICU stay were not included in the final results, reclaiming time in order to reach the desirable feeding levels.

### 2.5. Data Analysis

Categorical variables were presented as absolute (*n*) and relative frequencies (%), while the ENIs duration and nutrient intake were presented as the median (interquartile range, IQR) due to their non-normal distribution. Whether these variables were normally distributed was tested graphically, through a histogram and a P-P plot, and statistically, with a Shapiro–Wilk test. A Mann–Whitney U test was used to assess the median hours of ENIs among sex, age groups (adults versus older adults), BMI classification (normal versus overweight/obese), comorbidities, the need for mechanical ventilation, and energy intake (low versus high). The Wilcoxon signed-rank test was used to compare the median micronutrient intake to Dietary Reference Intake (DRIs). Beta coefficients and their corresponding 95% Confidence Intervals for the association of the daily average duration of ENIs (in hours) with their daily mean macro- and micro-nutrient intake were evaluated throughout multivariable linear regression analysis. We added three different models (Model 1. crude model, Model 2. multi-adjusted model for age and sex, Model 3. multi-adjusted model for age, sex, and comorbidity) for energy intake and each macro- and micronutrient intake separately to estimate the effects of the duration of ENI on these intakes. All models were tested for collinearity. Two-tailed hypothesis tests were performed. Stata SE 16.1 software (STATA Corp Ltd., 4905 Lakeway Drive, College Station, Texas 77845-4512, USA) was used for the statistical analysis, and a *p*-value ≤ 0.05 was regarded as statistically significant.

### 2.6. Ethical Principles

The research protocol was approved by the Institutional Ethics Committee of the University General Hospital “ATTIKON” (ΕΒΔ165/4-3-2019).

## 3. Results

**Figure 1:**
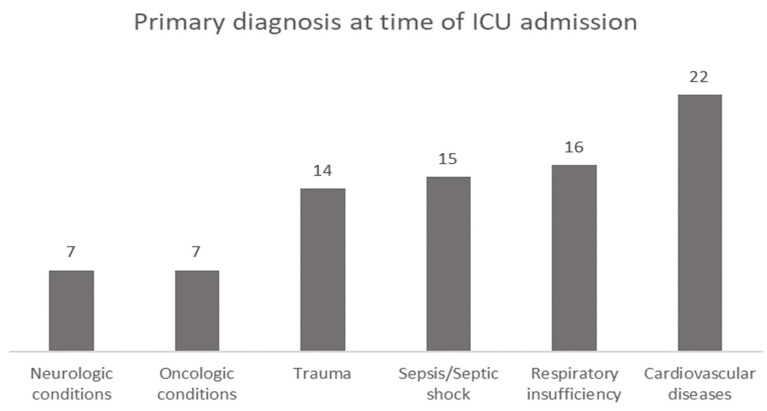
Primary diagnosis at the time of ICU admission.

A total of eighty-one patients who met the eligibility criteria were included in the cohort. Patients were classified according to the primary diagnosis at the time of ICU admission, with cardiovascular disease complications being in first place (27.2%), followed by respiratory insufficiency (19.8%), sepsis or septic shock (18.5%), trauma (17.3%), and neurologic or oncologic conditions (8.6%) (Figure 1).

Patients’ characteristics and median duration of ENIs by every single characteristic are presented in Table 1. The mean age was 64 ± 15 years of age, while 48.1% of patients were at least 65 years old (elderly). Most ICU patients were males (69.1%), and almost half of the sample was overweight or obese (48.8%). The vast majority (90.1%) required mechanical ventilation, and 46.9% had a burden of comorbidities, presented extensively in Supplementary Table S1. Mechanically ventilated patients had a higher duration of ENIs due to diagnostic procedures (*p* < 0.05) (Table 1). The mean energy intake was calculated to be 1037 ± 281 kcal/day, while 60.5% of patients covered less than 65% of their total energy needs (1751 ± 295 kcal/day). Patients with an energy intake <65% of their daily energy requirements had an increased median duration of ENIs by 1.3-fold compared with patients with a caloric intake >65% (*p* < 0.05). The median duration of ENIs (overall or due to diagnostic procedures, patient-related factors, or interventional procedures) did not differ significantly between age, sex, the presence of sepsis, and comorbidities. In addition, the mNUTRIC score was available for 45.7% of the sample, indicating that 56.8% were at a high nutritional risk (score > 5).

**Figure 2:**
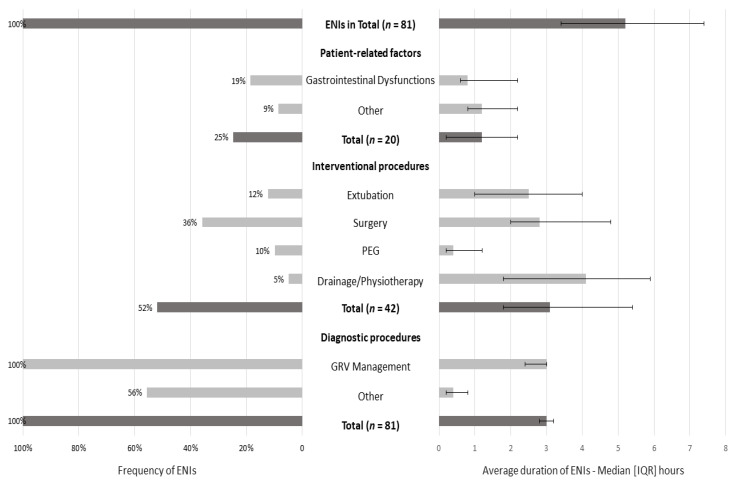
Frequency and average duration of ENIs for 5 days (3rd–7th day of ICU stay). Data are presented as numbers (%) and medians (IQR). Gastrointestinal dysfunctions included high GRV, diarrhea, and bowel ischemia. Other patient-related factors included pleural effusion and secretions. Other diagnostic procedures included body imaging techniques, such as CT scans, MRI, and abdominal ultrasounds (fasting for optimal visualization). The median duration of every ENI episode was calculated in the subsamples: interventional, diagnostic procedures, and patient-related factors (for instance, the median duration of ENIs due to diagnostic procedures was obtained from 45 patients).

ENIs were observed in all patients, with a median duration of 5.2 (3.4–7.4) hours (Figure 2). The most common cause was due to diagnostic procedures (100% of the sample), with an interrupted EN for a median duration of 3 h (2.8–3.2). The most common diagnostic procedure was GRV management: at least one time in all patients (median duration: 3 h (2.4–3)). The second most common factor was interventional procedures (52% of patients; median duration: 3.1 (1.8–5.4) hours), with surgery as the primary interventional procedure (36% of patients; median duration: 2.8 (2–4.8) hours), and the last was patient-related factors (25% of patients; median duration: 1.2 (0.2–2.2) hours).

A gap between the macro- and micronutrient intake and the DRIs was observed, since the median administration was significantly less in all nutrients, except for manganese, compared with DRIs (all *p*-values ≤ 0.01) (Table 2). In all patients, the daily protein (median daily intake (MDI) = 0.43 g/kg), arginine (MDI = 0.37 g), and vitamin D (MDI = 7.76 μg) intakes were lower compared with DRIs, with the protein intake barely reaching 0.43 ± 0.3 g/kg. In more than 70% of patients, the daily intake of carbohydrates (MDI = 100 g), omega-3 FA (MDI = 326 mg), vitamin E (MDI = 10.3 mg), and selenium (MDI = 47.6 μg) was also decreased in correlation with DRIs. More than 60% of the sample received insufficient amounts of vitamin A (MDI = 588 μg), zinc (MDI = 8.29 mg), and vitamin C (MDI = 65.3 mg) compared with the DRIs.

In Table 3, we conducted univariable and multivariable linear regression analysis to estimate the impact of the ENIs duration on the energy and macro- and micronutrient intake. We added three different models separately for the energy intake and each macro- and micronutrient intake. The interpretation of the linear regression models showed that every lost hour of enteral nutrition was significantly associated with macro- and micronutrient deficits in ICU patients (all *p*-values < 0.05) in both crude and adjusted models. For every lost hour of feeding, a mean of 60.3 kcals, 7.5 g of carbohydrates, 2.75 g of protein, and 2.33 g of fat were lost, respectively, according to multi-adjusted models (*p*-values < 0.001). Similarly, for micronutrients and antioxidants, every lost hour was connected with a significant quantitative loss of 55.5 μg RE/day for vitamin A, 3.61 μg/day for selenium, 0.13 mg/day for manganese (*p*-values < 0.001), 0.81 μg/day for vitamin D, 0.59 mg/day for zinc, 83.4 mg/day for omega-3 fatty acids, and 0.07 g/day for arginine (*p*-values < 0.05).

## 4. Discussion

### 4.1. Causes, Frequency, and Duration of ENIs

The current study sheds light on the causes, frequency, and duration of ENIs, while macro- and micronutrient deficits in ICU patients were highlighted as a subsequent effect. The most frequent causes of ENI episodes were diagnostic procedures, specifically GRV monitoring (100% of the sample, median duration: 3 (2.4–3) h). Mechanically ventilated patients had the longest ENIs due to GRV management. Documentation and medical orders were frequently missing, and a nutrition support team was absent, especially the dietitian. The interruption of EN as an unavoidable reality led to substantive energy and protein deficits. Finally, for every lost hour of feeding, a significant number of calories and nutrients was estimated to be lost (i.e., 60.3 kcals and 2.75 g of protein).

Interestingly, the reasons for ENIs in the present study seem to be different from those published in other papers. The primary cause we identified was GRV monitoring, a common practice in ICU patients. Onuk et al., in a recent study, mentioned that radiological procedures were the most common reason for ENIs, with the longest ENIs being caused by tube feeding malfunctions during the first week of the ICU stay [32]. These findings were confirmed in Peev’s and O’Meara’s publications (ninety-four and fifty-nine mechanically ventilated patients, respectively) in the United States, highlighting that feeding tube problems are the most common cause of ENIs [33,34]. Airway management issues were reported in the literature as another common cause leading to ENI episodes [35,36], while the hemodynamic instability of patients was the main reason for ENIs in Salciute-Simene’s publication [2]. In our study, the median length of ENIs was 5.2 h, which is consistent with Uozumi’s findings [36] and significantly lower than that found by Salciute-Simene (median duration of ENIs of 12 h) [2]. Lee et his colleagues revealed a total duration of feeding interruptions for the whole ICU stay of 24.5 h, mainly due to procedural-related, potentially avoidable reasons [37].

### 4.2. Protein and Caloric Deficits

The median daily protein intake did not exceed 0.43 ± 0.3 gr of the actual body weight, whereas the ESPEN recommendation is 1.3 g/kg for adjusted body weight/d (limitations included in the same section) [14], and the ASPEN expert consensus suggests a daily intake in the range of 1.2–2.0 g/kg. Until now, evidence for high protein intake and its efficacy remains controversial [38]. Post hoc analyses of the data from the Early Parenteral Nutrition Completing Enteral Nutrition in Adult Critically Ill Patients (EPANIC) study indicate that protein intake is associated with unfavorable outcomes [39].

Our results demonstrate that patients received lower amounts of protein but match with current data indicating that ICU patients receive an average of 0.6 g/kg/day for the first two weeks [17,40,41,42,43,44,45,46,47].

The energy deficit for the daily caloric goal was 38.7% (±19.7). It was surprising that the prevalence of energy deficits was one of the highest in the literature; Adamo et al. reported caloric deficits of 24% [35], Uozumi et al. reported 11.5% [36], and Kıter et al. reported 17.1%, respectively [48]. It should be noted that, despite the importance of adequate nutritional support in critically ill patients, it is well-established that the concept of ‘permissive underfeeding’ may be associated with clinical benefits in these patients, while underfeeding for a short period is not accompanied by adverse events [49]. Nonetheless, the extent of underfeeding (trophic to 70% of EN) during the first week of admission may differ for each patient and is not yet established [7]. Two meta-analyses found that a high-energy intake may increase complications in ICU patients who are not malnourished, and hypocaloric feeding is associated with better clinical outcomes [42,43]. Some may argue that the lower amount of feeding associated with ENIs will not benefit critically ill patients (even those with low nutritional risk). However, until now, the optimal calorie and protein intake remains unknown [50].

### 4.3. GRV Management

The restriction of ENIs, attempted for avoidable purposes, seems to be of vital importance. Despite GRV management being a common practice in ICU patients and the main reason for ENIs in our study, in another study, the removal of GRV monitoring resulted in a significantly increased EN provision [51]. According to Poulard and his colleagues, GRV monitoring may impede the EN provision, leading to underfeeding, by causing unnecessary ENIs. Their research team concluded that stopping EN delivery when GRV reaches a randomly determined cutoff level is not justified by scientific evidence and does not prevent the risk of ventilator-associated pneumonia [52]. Globally, some ICUs have stopped routinely measuring GRV, except for indicated cases (nausea, vomiting, or distension), whilst others continue to engage in protocol-driven GRV checks in their patients due to the lack of appropriate guidelines [36,53].

### 4.4. Micronutrient and Antioxidant Intake

**Figure 3:**
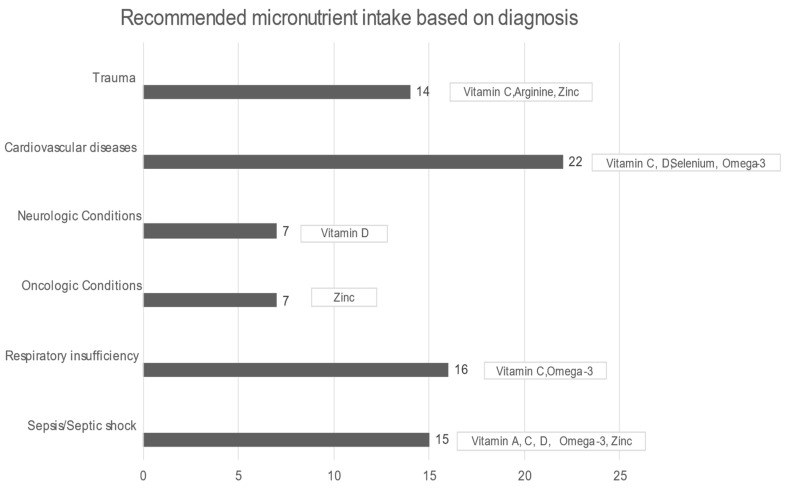
Recommended micronutrient intake based on diagnosis [8,14,27,47].

In all patients, the median daily intake was significantly less in all micronutrients, except for manganese, compared with DRIs (all *p*-values < 0.01). Starting with arginine, in healthy situations, it is considered a nonessential amino acid, but in critical illness, it becomes an essential component for protein synthesis and immunomodulation. While arginine supplementation in sepsis remains controversial, on the other hand, it is recommended for wound healing and postoperative surgical and severe trauma patients [54]. A total of 92.6% of the sample had a significantly lower median daily intake of omega-3, while the International Society for the Study of FA and Lipids recommends a daily intake of 500 mg of EPA (eicosapentaenoic acid, EPA) and (docosahexaenoic acid, DHA) DHA (for healthy humans). In ICU patients, omega-3 could offer a therapeutic advantage in conditions such as ARDS, sepsis, and even after major surgery or trauma, while it potentially alleviates inflammatory procedures, reduces hypertriglyceridemia, and benefits patients with heart disease and atrial fibrillation. The current nutrition guidelines recommend diets rich in anti-inflammatory lipids, such as omega-3 FAs, for patients with ARDS [14]. Furthermore, EPA and DHA were associated with reduced mortality in patients with sepsis, especially those with gastrointestinal dysfunction [55], and formulas enriched with omega-3 were strongly recommended by ESPEN [18]. The selenium intake was also below the proposed DRIs levels; selenium is required for the synthesis of the amino acid selenocysteine, an essential component of at least 25 selenoproteins, with antioxidant and redox activity in human tissues. Burn and major trauma patients and patients receiving renal replacement therapy and cardiac surgery have increased oxidative stress and high losses of selenium, therefore requiring increased amounts of selenium [27]. A gap between the desirable and realistic median daily intake also existed in the case of vitamin C, the most potent antioxidant, which alleviates oxygen radicals’ production and recycles other antioxidants. It participates in the biosynthesis of neurotransmitters, peptide hormones, cortisol, and collagen while protecting the endothelium and maintaining vasodilation and endothelial barrier function [27]. Vitamin D was significantly lower than the DRIs directives as well. In acute illness, vitamin D supplementation should be provided at least according to the DRIs indications determined for healthy subjects, since, in the presence of inflammation, a significant drop in plasma levels is noticed at the expense of vitamin D (indicatively when C- reactive protein (CRP) > 40 mg/L) [27]. In addition, the median daily intake of vitamin A was remarkably lower than DRIs. Vitamin A plays a crucial role in the immune system, and its deficiency may cause an imbalance between pro- and anti-inflammatory factors. Inflammation reduces the absorption of vitamin A and increases its requirement, thus contributing to inadequacy [27]. The median zinc intake was also insufficient compared with DRIs. Zinc represents an important part of the antioxidant defense system. Zinc malabsorption may occur in patients with short bowel syndrome, bariatric surgery, cystic fibrosis, chronic pancreatitis, or inflammatory bowel disease. Increased urinary loss may be present in hypercatabolic conditions, such as burns, trauma, and sepsis, in renal disease, and in alcoholism, while prolonged renal replacement therapy may cause deficiencies [27] (Figure 3).

### 4.5. The role of Dietitians

Whereas ICUs follow feeding protocols, evidence continually suggests that they are not sufficient to prevent nutritional deficits, and, thus, individualized nutrition support and care are better provided when a critical care dietitian is involved in the multidisciplinary team [56,57].

### 4.6. Strengths and Limitations

To our knowledge, this is the first study to evaluate the impact between ENIs and antioxidants deficits. The novelty of our research in this field emphasizes the necessity of feeding protocol remodeling in ICUs to reduce the risk of malnutrition-related complications. Targeted micronutrient supplementation will lead to better outcomes and a reduction in the average length of stay.

This cohort study has some limitations. The energy needs of the patients were calculated at 25 kcal/kg (Ideal Body Weight) of energy and 1.2 g/kg of proteins daily, independent of the stage and disease progression. On the one hand, body weight was used to estimate protein goals, not adjusted for fluid retention or positive fluid balance, which could lead to the overestimation of protein goals. On the other hand, all patients received significantly less protein than recommended, with an average daily intake equal to one-third of the goal, something that could not change even with the presence of oedemas. Consequently, we hypothesized that this could be attributed to the combination of under-prescribing by physicians and the limited involvement of dieticians in the daily monitoring and evaluation of critical care patients. This is a single-center non-controlled study, leading to a potential bias in the results due to the lack of a control group. However, the results of this observational study could be used as pilot data that could inform future well-designed studies. Finally, due to the pandemic, the entrance in the ICU was forbidden, and all studies stopped; hence, the sample size of our study was smaller than calculated (81 from 93). Nevertheless, we found highly significant differences in the energy, protein, and nutrient intakes of our subjects compared with the recommended levels and demonstrated a significant impact of ENIs, indicating a major problem related to micronutrient and antioxidant deficiencies in ICU patients.

## 5. Conclusions

The main cause of ENIs was diagnostic procedures—specifically, GRV monitoring. The ENIs resulted in approximately 60.5% of patients failing to meet the calculated caloric requirements. Similarly, the results revealed that ENI episodes could lead to substantial protein and antioxidant deficits. Even though these results were suboptimal compared with the guidelines, they are keeping up with general international trends.

Uozumi et al. supported the development of a feeding protocol for ENIs management that could possibly prevent energy deficits at an early stage [28]. Based on the findings of our study, it is proposed that guidelines should include an interruption-minimizing protocol so as to reduce the missing hours and improve clinical outcomes. It is highly important that dietitians should participate and be part of the multidisciplinary team, promoting the regular daily assessment of the nutritional status in the ICU. A holistic therapeutic approach should comprise nutritional assessment and treatment in order to diminish metabolic disturbances and prevent poor outcomes.

## Figures and Tables

**Table 1:** Average duration of ENIs for 5 days (3rd–7th day of the ICU stay) according to the baseline characteristics of the cohort (*n* = 81).

Baseline Characteristics of the Cohort	*n* (%)	Median Hours (IQR)			
		Diagnostic Procedures	Patient-Related Factors	Interventional Procedures	Total
**Age**					
<65 years old	42 (51.9)	3 (2.8–3.2)	0 (0–0)	1.3 (0–3.2)	5.7 (3.6–7.4)
≥65 years old	39 (48.1)	3 (2.6–3.4)	0 (0–0.6)	0 (0–3)	5 (3–8)
**Sex**					
Males	56 (69.1)	3 (2.8–3.2)	0 (0–0)	0.2 (0–3.1)	4.9 (3.2–7.5)
Females	25 (30.9)	3 (2.8–3.6)	0 (0–0.8)	1 (0–3.2)	5.8 (3.6–7.4)
**BMI**					
Normal	41 (51.2)	3 (2.6–3.2) *	0 (0–0)	0.4 (0–3.2)	5.2 (3.4–7.4)
Overweight/Obese	39 (48.8)	3 (3–3.6) *	0 (0–0.4)	0 (0–3.2)	5.6 (3.6–8.4)
**Septic patients**					
Yes	15 (14.8)	3 (2.8–3.4)	0 (0–2.2)	0 (0–2.2)	5.6 (3.2–7.4)
No	66 (85.2)	3 (2.8–3.2)	0 (0–0)	0.4 (0–4)	5 (3.4–8.4)
**Mechanically Ventilated Patients**					
Yes	73 (90.1)	3 (2.8–3.4) *	0 (0–0.4)	0.4 (0–3.2)	5.4 (3.4–7.4)
No	8 (9.9)	2.6 (1.7–2.9) *	0 (0–0)	0 (0–2.4)	3.9 (3.3–8.2)
**With comorbidities**					
Yes	38 (46.9)	3 (3–3.2)	0 (0–0)	0.1 (0–2.4)	4.9 (3–7)
No	43 (53.1)	3 (2.6–3.4)	0 (0–0.4)	1 (0–4.2)	6 (3.6–8.4)
**Energy intake**					
<65% of MEE	49 (60.5)	3 (2.6–3.4)	0 (0–0.6)	1 (0–4.6)	6 (3.8–8.4) *
≥65% of MEE	32 (39.5)	3 (3–3.2)	0 (0–0)	0 (0–2.2)	4.4 (3–5.8) *

The median duration of any ENI episode was calculated for all patients in every group (* *p* < 0.05;). The Mann–Whitney U test was used to compare the duration of ENIs among the baseline characteristics of the cohort. Abbreviations: Measured Energy Expenditure (MEE); Enteral Nutrition Interruptions (ENI); Interquartile Range (IQR).

**Table 2:** Median daily intake of macro- and micronutrients compared with ESPEN guidelines for adults (*n* = 81).

Nutrient	Median Daily Intake	DRIs	*p*-Value	Patients with Intake <DRIs, *n* (%)
Carbohydrates (g/day)	100 (79–117)	130	<0.001	68 (84.0)
Protein (g/kg)	0.43 (0.32–0.59)	1.2 g/kg	<0.001	81 (100)
Fat (g/day)	26.5 (20.6–35.9)	ND	-	-
Vitamin A (μg RE/day)	588 (480.8–885.6)	900 males 700 females	<0.001	55 (67.9)
Vitamin C (mg/day)	65.3 (50.7–99.2)	90 males 75 females	0.010	53 (65.4)
Vitamin E (mg α-TE/day)	10.3 (8.6–13.7)	15	<0.001	64 (79.0)
Selenium (μg/day)	47.6 (39.1–56.3)	55	<0.001	57 (70.4)
Manganese (mg/day)	1.93 (1.54–2.35)	2.3 males 1.8 females	0.161	52 (64.2)
Vitamin D (μg/day)	7.76 (5.99–9.61)	15 for <70 years old 20 for >70 years old	<0.001	80 (98.8)
Zinc (mg/day)	8.29 (6.45–10.1)	11 males 8 females	<0.001	55 (67.9)
Omega-3 fatty acids (mg/day)	326 (202–451)	500	0.002	64 (79.0)
Arginine (g/day)	0.37 (0–1.02)	5–7	<0.001	81 (100)

The Wilcoxon signed rank test was used to compare the patients’ nutrient intake with DRIs. Abbreviations: Dietary Reference Intakes (DRIs); Not determined (ND); Grams (g); Day (d); Milligrams (mg); Micrograms (μg); Retinol Equivalents (RE); Alpha-Tocopherol Equivalents (α-TE); Fatty Acids (FA); The European Society for Clinical Nutrition and Metabolism (ESPEN).

**Table 3:** The β coefficients and their corresponding 95% confidence interval of the linear regression models for the association of the daily average duration of ENIs (in hours) (independent variable) with their daily mean macro- and micronutrient intake (dependent variables) (*n* = 81).

Dependent Variable	Model 1—Crude Model	Model 2 ^a^	Model 3 ^b^
	β (95% CI)	β (95% CI)	β (95% CI)
Energy (kcal/day)	−60.9 (−79.2, −42.6) **	−61.2 (−79.6, −42.9) **	−60.3 (−78.9, −41.7) **
Carbohydrates (g/day)	−7.46 (−9.41, −5.48) **	−7.44 (−9.43, −5.45) **	−7.5 (−9.53, −5.48) **
Protein (g/day)	−2.79 (−3.63, −1.95) **	−2.78 (−3.63, −1.94) **	−2.75 (−3.61, −1.89) **
Fat (g/day)	−2.37 (−3.37, −1.36) **	−2.37 (−3.39, −1.35) **	−2.33 (−3.36, −1.29) **
Vitamin A (μg RE/day)	−57.4 (−83.4, −31.4) **	−57.6 (−84.2, −31.1) **	−55.5 (−82.3, −28.7) **
Vitamin C (mg/day)	−9.6 (−19.3, 0.2)	−9.6 (−19.5, 0.39)	−9.0 (−19.3, 1.1)
Vitamin E (mg α-TE/day)	−2.03 (−4.54, 0.49)	−2.02 (−4.58, 0.55)	−1.91 (−4.52, 0.69)
Selenium (μg/day)	−3.62 (−4.61, −2.64) **	−3.6 (−4.6, −2.6) **	−3.614 (−4.63, −2.6) **
Manganese (mg/day)	−0.129 (−0.183, −0.075) **	−0.129 (−0.184, −0.074) **	−0.127 (−0.183, −0.071) **
Vitamin D (μg/day)	−0.799 (−1.042, −0.556) **	−0.8 (−1.041, −0.56) **	−0.805 (−1.05, −0.561) **
Zinc (mg/day)	−0.596 (−0.788, −0.404) **	−0.588 (−0.783, −0.394) **	−0.594 (−0.791, −0.396) **
Omega-3 FA (mg/day)	−87.3 (−152.5, −22.1) **	−86.6 (−153, −20.1) *	−83.4 (−150.6, −15.9) *
Arginine (g/day)	−0.065 (−0.129, −0.001) *	−0.062 (−0.126, 0.003)	−0.068 (−0.133, −0.004) *

In all multivariable linear regression models, the daily average duration of ENIs (hours) is the independent variable (* *p* < 0.05; ** *p* < 0.001). ^a^ Adjusted for age and sex; ^b^ Adjusted for age, sex, and comorbidity. Abbreviations: 95% confidence interval (95% CI); Grams (g); Day (d); Milligrams (mg); Micrograms (μg); Retinol Equivalents (RE); Alpha-Tocopherol Equivalents (α-TE); Fatty Acids (FA).

## Data Availability

The data used in the analysis are available from the corresponding author upon reasonable request.

## Supplementary Materials

The following supporting information can be downloaded at: https://www.mdpi.com/article/10.3390/nu15040917/s1, Table S1: Reasons for the inability to feed and comorbidities in ICU patients (*n* = 81).

## Abbreviations

APACHE	Acute Physiology Age Chronic Health Evaluation
ARDS	Acute Respiratory Distress Syndrome
BMI	Body Mass Index
DHA	Docosahexaenoic acid
DRI	Dietary Reference Intake
EN	Enteral Nutrition
ENI	Enteral Nutrition Interruption
EPA	Eicosapentaenoic acid
ESPEN	European Society for Clinical Nutrition and Metabolism
FA	Fatty acids
GRV	Gastric Residual Volume
HDL	High-Density Lipoprotein
ICU	Intensive Care Unit
IQR	Interquartile Range
LDL	Low-Density Lipoprotein
MDI	Median Daily Intake
mNUTRIC	Modified Nutrition Risk in Critically ill
PEG	Percutaneous Endoscopic Gastrostomy
PN	Parenteral Nutrition
PUFA	Polyunsaturated Fatty Acids
SOFA	Sequential Organ Failure Assessment
