# Supplementary material for: Factors Associated with Interruptions of Enteral Nutrition and the Impact on Macro- and Micronutrient Deficits in ICU Patients
Source: Nutrients. 2023 Feb 11;15(4):917. doi: 10.3390/nu15040917 (PMC9959226; doi:10.3390/nu15040917)
Supplement: Supplementary file 1 [file nutrients-15-00917-s001.zip › nutrients-2142518-supplementary.pdf]

nutrients

Supplementary Materials

Supplementary Table S1. Reasons for inability to feed and comorbidities in ICU patients (N=81).

**Reasons for inability to feed**	**N (%)**
Patients under sedation	8 (9.88)
Patients in mechanical ventilation under sedation	73 (90.12)
**Comorbidities (N=43)**	
Diabetes mellitus	6 (13.95%)
Diabetes mellitus & hypertension<br>(3 of them with renal disease)	17 (39.53%)
ARDS & hypertension	5 (11.63%)
ARDS & Influenza	1 (2.33%)
Infections	9 (20.93%)
Gastrointestinal disorders	3 (6.98%)
Chronic Obstructive Pulmonary Disease (COPD)	2 (4.65%)
